# Multiligament knee injury patients with limited access to post‐operative rehabilitation exhibit similar patient‐reported outcomes: A retrospective cohort study

**DOI:** 10.1002/jeo2.70245

**Published:** 2025-04-22

**Authors:** Shane S. Korber, Amir Fathi, Ioanna K. Bolia, Cailan L. Feingold, Eric H. Lin, Samantha A. Solaru, Neilen Benvegnu, Joseph N. Liu, George F. Rick Hatch

**Affiliations:** ^1^ Department of Orthopaedic Surgery Keck School of Medicine of USC Los Angeles California USA

**Keywords:** access to care, knee dislocation, multiligament knee injury, post‐operative rehabilitation, rehabilitation outcome, social determinants of health

## Abstract

**Purpose:**

Multiligament knee injuries (MLKI) are typically high‐energy traumatic injuries requiring surgical reconstruction and extensive post‐operative rehabilitation. This study aimed to examine differences in outcomes of patients with different access to post‐operative rehabilitation following multiligament knee reconstruction (MLKR). We hypothesize that patients with limited access to rehabilitation will demonstrate worse outcomes.

**Methods:**

Patients who sustained an MLKI between 2007 and 2019 and who underwent MLKR by a single surgeon were retrospectively identified and invited to participate. Patients less than 1 year post‐operative were excluded. Data recorded included patient demographics, intraoperative procedure performed, patient access to rehabilitation after surgery (limited versus full access), and multiple post‐operative patient‐reported outcome measures (PROMs). A comparison of PROMs was performed between patients with limited versus full access to rehabilitation using descriptive statistics (STATA). The level of statistical significance was set at *p* < 0.05.

**Results:**

Eighty‐three patients (17.4% female) met the inclusion criteria and had accessible rehabilitation data. Of those, 69 (83.1%) patients had full access to rehabilitation, and 14 patients (16.9%) had limited access to post‐operative rehabilitation. There was no difference in mean follow‐up time (2.6 and 2.2 years, respectively, *p* = 0.96) or baseline patient characteristics. Patients with limited access to post‐operative rehabilitation had significantly worse Patient‐Reported Outcome Measurement Information System (PROMIS) Pain (*p* = 0.021) and PROMIS Physical Function (*p* = 0.023) scores compared to patients with full access to rehabilitation. PROMIS mobility, Lysholm and multiligament quality of life (MLQOL) measures were not significantly different.

**Conclusion:**

Patients who underwent MLKI reconstruction with limited access to rehabilitation demonstrated worse PROMIS pain and physical function scores than those with full access to rehabilitation in the short term. However, these differences do not meet minimum clinically important difference values, suggesting similar outcomes. Other PROMs, such as PROMIS mobility, Lysholm and MLQOL scores, were similar between patients with and without full access to rehabilitation.

**Level of Evidence:**

Level III, retrospective cohort study.

**Abbreviations**
ACL: anterior cruciate ligament
KD: knee dislocation
LCL: lateral collateral ligament
MCID: minimal clinically important difference
MCL: medial collateral ligament
MLKI: multiligament knee injury
MLKR: multiligament knee reconstruction
MLQOL: multiligament quality of life
PCL: posterior cruciate ligament
PROM: patient‐reported outcome measure
PROMIS: PatIent‐Reported Outcomes Measurement Information SyStem
ROM: range of motion
TKA: total knee arthroplasty

## INTRODUCTION

Multiligament knee injuries (MLKI) are a traumatic, life‐altering and highly variable set of injuries that pose a challenge to the treating surgeon [12, 19, 31, 35]. The injury profiles that fall under the category of MLKI range from injury to at least two of the major knee ligaments up to open fracture and dislocation with concomitant neurovascular injury [9, 24]. Surgical treatment of these injuries is required to restore stability to the knee and prevent further chondral injury [8, 36]. Most patients undergoing reconstruction achieve excellent knee function [28]. Considerations for the surgeon include timing of the operation, staged versus non‐staged treatments, and post‐operative rehabilitation protocols [30, 33]. The rare, heterogeneous, and traumatic nature of MLKIs makes them difficult to study in well‐controlled, prospective trials, leading to a paucity of literature guiding decision‐making [24].

Post‐operative course and rehabilitation after surgery for a ligamentous injury of the knee play an important role in the treatment process. For MLKIs, the exact post‐operative protocol varies based on surgeon preference as well as injury severity [20, 26, 44]. Many surgeons stratify weight‐bearing progression based on which ligamentous structures are injured [10, 25]. In other orthopaedic sports procedures like anterior cruciate ligament (ACL) reconstruction and meniscal and osteochondral allograft transplantation, literature has demonstrated that supervised post‐operative rehabilitation, as well as patient compliance with these protocols, improve outcomes [7, 16, 34, 37, 45].

Access to and compliance with rehabilitation is affected by multiple factors, including socioeconomic status and other sociodemographics like education level, insurance status, and proximity to rehabilitation centres [3]. Past studies have shown that patients from lower socioeconomic backgrounds often have worse outcomes across many common orthopaedic procedures [2, 14, 18]. Additionally, a lower level of education is also associated with worse outcomes in orthopaedic procedures [22, 47]. While literature supporting the role of access to rehabilitation is strong for other orthopaedic sports procedures, there is a lack of research investigating outcomes in patients treated surgically for MLKI with full access to rehabilitation compared to those with limited access to rehabilitation.

We hypothesize that patients with full access to rehabilitation would have improved patient‐reported outcomes. The purpose of this study was to compare patient‐reported outcome measures (PROMs) in patients with a structured, regimented rehabilitation protocol versus in patients with limited access to rehabilitation treated by the same surgeon.

## METHODS

This study was approved by the Institutional Review Boards of the University of Southern California (#HS‐17‐00301). A retrospective review was performed, and patients sustaining MLKI who underwent multiligament knee reconstruction (MLKR) by a single sports medicine fellowship‐trained surgeon at one of two hospitals between 2007 and 2020 were identified. One group of patients had access to a regimented physical therapy programme, and the other group of patients had very limited access to rehabilitation. MLKI was defined as two of the four major ligaments injured (ACL, posterior cruciate ligament [PCL], medial collateral ligament [MCL] and lateral collateral ligament [LCL]).

Inclusion criteria were (1) surgical treatment of MLKI, (2) at least 1 year since reconstructive surgery, (3) at least 16 years of age at the time of injury (4) and willingness to participate after the informed consent process. Patients were excluded if they underwent previous surgery on the affected knee, underwent nonoperative treatment for MLKI, were unable to be contacted for post‐operative follow‐up, had incomplete data, were neither English nor Spanish speaking, or refused to participate.

### Patient evaluation and data collection

In April 2020, patients were contacted by telephone by one of three authors (S.K., N.B. and A.F.). They received a scripted description of the study and were asked to participate in the informed consent process before the administration of our MLKI‐validated instruments. No preoperative outcomes were collected. Patient demographic information, including gender, age, body mass index (BMI), procedure performed and access to rehabilitation, were collected via chart review.

### Data collection instruments

The multiligament quality of life (MLQOL) questionnaire instrument was validated for MLKIs in 2014 and was designed to be more responsive to unique elements of MLKIs, including vascular and neurologic injuries [42]. The instrument has four domains: Physical Impairment, Emotional Impairment, Activity Limitations and Societal Involvement, each of which has 13 associated questions [4]. The Lysholm score has also been validated for use in MLKIs [42].

The universal Patient‐Reported Outcomes Measurement Information System (PROMIS) computer adaptive testing (CAT) was also utilized as it has been validated for MLKIs [43]. The PROMIS CAT tool was introduced to create a universally applicable instrument that adapts to the patient's antecedent responses, resulting in both variable questions and lengths. The Pain, Physical Function and Mobility components of this score correlate well with existing measures (including the MLQOL questionnaire and Lysholm score) for MLKIs [43].

### Surgical technique and post‐operative rehabilitation

The senior author followed the same operative technique for both those with and without access to rehabilitation. After general anaesthesia induction, the patients' ACL, PCL, LCL and MCL underwent stress testing with fluoroscopy, which determined which structures would be addressed surgically. Ligament injury location (i.e., avulsion vs. mid‐substance rupture) and overall tissue quality were assessed on MRI and intra‐operatively determined whether the structures would be repaired or reconstructed.

A structured rehabilitation programme was implemented post‐operatively for patients with full access to the rehabilitation group (Table 1). Patients were braced in full extension with touch‐down weight‐bearing for the first 4 weeks. Quadriceps strengthening was initiated immediately. Cryotherapy was performed 6–8 times per day to reduce swelling. Over Weeks 5–8, weight‐bearing progressed to 75% with a goal of full range of motion (ROM) defined as 0–135°. Quadriceps strengthening was continued, while open‐chain hamstring strengthening was avoided. Straight running was resumed at 9 months.

**Table 1:** Comparison of post‐operative rehabilitation protocols between full and limited access to rehabilitation.

	Full access rehabilitation protocol	Limited access rehabilitation protocol
Protocol Initiation	Immediately post‐operative	2 weeks post‐operative (based on therapist availability)
Weeks 1–4	Braced in full extension	Knee immobilizer (Weeks 0–2) → hinged knee brace obtained in clinic
	Toe‐touch weight‐bearing	Toe‐touch weight‐bearing
	Quadriceps strengthening	Quadriceps strengthening
	Cryotherapy 6–8×/day	Goal ROM: 0–90°
Weeks 5–8	Weightbearing progressed to 75% Goal ROM: full (0–135°) Continued quadriceps strengthening	Toe‐touch weight bearing (until Week 6) Weightbearing progression varied

Abbreviation: ROM, range of motion.

The rehabilitation programme for those with limited access was less consistent, with the programme varying considerably based on therapist availability (Table 1). The rehabilitation process was supervised via clinic visits with the surgeon. However, physical therapy staff availability and the ability of the patient to remain compliant made further consistency challenging. Patients did not start supervised therapy until 2 weeks due to limited therapist staffing. Patients were placed in a knee immobilizer for the first 2 weeks until they were able to obtain a hinged knee brace in the clinic. In general, patients were made toe‐touch weight‐bearing for the first 6 weeks, with a focus on quadriceps strengthening. ROM was typically set to 0–90° for the first 4 weeks, with progression based on individual patient requirements.

### Statistics

Statistics were performed with Stata (v13.0; StataCorp). Shapiro–Wilk test was used to check for normal or non‐normal distribution of data. Fisher exact test was used to compare knee dislocation (KD) class distributions between the patient groups. Mann–Whitney *U* tests were performed for comparison of continuous variables if at least one group had a non‐normal distribution with the level of statistical significance set at *p* < 0.05. If both groups being compared were normally distributed, then two‐tailed *t* tests were performed with a level of statistical significance set at *p* < 0.05.

## RESULTS

**Figure 1:**
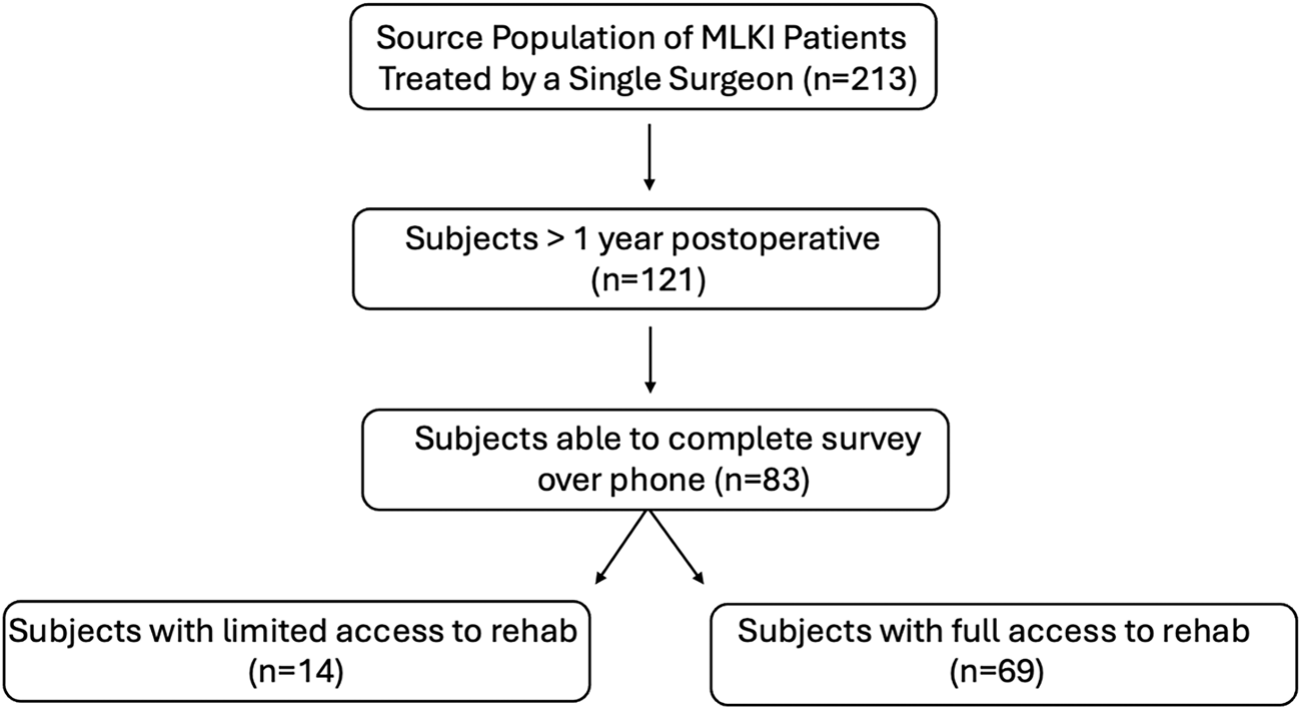
Participants numbers at each stage of study. MLKI, multiligament knee injury.

After the identification of patients who underwent surgical management of MLKI, 83 met the inclusion criteria (Figure 1). Access to rehabilitation data was available for all 83 patients. Of these, 69 patients (17.4% female, average age 36.5 ± 17 years old) had full access to rehabilitation and 14 (17.6% female, average age 32.7 ± 3 years old) had limited access to post‐operative rehabilitation.

The mean follow‐up time was similar between the two groups (2.6 ± 0.3 years for full access and 2.2 ± 0.6 years for limited access, *p* = 0.96). Of patients with full access to rehabilitation, five (7.2%) had an external fixator before reconstruction. No patients from the limited access to rehabilitation group had an external fixator. For patients with full access to rehabilitation, 29 (42%) were of Schenk KD Class I, 10 (15%) were of KD Class II, 23 (33%) were of KD Class III and 7 (10%) were of KD Class IV. For patients with limited access to rehabilitation, 3 (21%) were of KD Class I, 11 (79%) were of KD Class III and none were KD Classes II or IV. The distribution of KD classes between groups was significantly different (*p* = 0.023). Table 2 demonstrates baseline patient demographics, which were similar between the two groups.

**Table 2:** Comparison of key patient demographics between patients.

Patient demographics	Full access rehabilitation (*n* = 69)	Limited access rehabilitation (*n* = 14)	*p* **value**
Age	36.50 ± 1.7	32.7 ± 3.0	0.360
Time between surgery to survey (days)	965.4 ± 111.3	830.2 ± 209.3	0.613
Follow‐up (days)	500.1 ± 56.4	507.7 ± 209.4	0.9610
Gender: (F)	12/69	3/14	0.720
BMI	30.1 ± 1.1	27.5 ± 1.1	0.317

Abbreviation: BMI, body mass index.

By the Shapiro–Wilk test, patients with full access to rehabilitation demonstrated non‐normal distribution for all PROMs (*p* < 0.05), while patients with limited access to rehabilitation demonstrated normal distributions for all PROMs (*p *> 0.05). Therefore, comparisons between PROMs were conducted using the Mann–Whitney *U* test. Patients with full access to rehabilitation achieved significantly superior scores on all three PROMIS measurements (Table 3): lower PROMIS Pain (52 ± 1.2 vs. 58.7 ± 2, *p* = 0.021) and higher PROMIS Physical Function (46.3 ± 1.3 vs. 38.3 ± 2.3, *p* = 0.023) scores compared to patients with limited access to rehabilitation. The PROMIS Mobility (45.3 ± 1.2 vs. 39.7 ± 2.5, *p* = 0.078) and MLQOL categories failed to reach statistical significance: MLQOL Physical Impairment (40 ± 2.9 vs. 41.8 ± 6.7, *p* = 0.696), MLQOL Emotional Impairment (46.8 ± 3.4 vs. 50.7 ± 6.8, *p *= 0.737), MLQOL Activity Limitations (37 ± 3.1 vs. 45.4 ± 6.4, *p* = 0.269) and MLQOL Societal Involvement (42.8 ± 3.3 vs. 50.9 ± 5.3, *p* = 0.274). The Lysholm score also failed to reach statistical significance between the two groups (61.4 ± 2.8 vs. 58.6 ± 7.2, *p* = 0.769).

**Table 3:** Comparison of various patient‐reported outcome measures between patients with full versus limited access to rehabilitation.

Patient‐report outcome measure	Full rehabilitation access (*n* = 69)	Limited rehabilitation access (*n* = 14)	*p* **value**
Promis pain	52.0 ± 1.2	58.7 ± 2.0	**0.021**
Promis physical function	46.3 ± 1.3	39.3 ± 2.3	**0.023**
Promis mobility	45.3 ± 1.3	39.7 ± 2.5	0.078
MLQOL physical impairment	40.0 ± 2.9	41.8 ± 6.7	0.696
MLQOL emotional impairment	46.8 ± 3.4	50.7 ± 6.8	0.737
MLQOL activity limitation	37.0 ± 3.1	45.4 ± 6.4	0.269
MLQOL societal involvement	42.8 ± 3.3	50.9 ± 5.3	0.274
Lysholm	61.4 ± 2.8	58.6 ± 7.2	0.769

*Note*: Bold values are statistical significant.

Abbreviation: MLQOL, multiligament quality of life.

A comparison of PROMs by KD class subgroup can be found in Supporting Information: Table. PROMIS Pain and PROMIS Physical Function were significantly worse in KD three patients with limited rehabilitation access (*p* = 0.0239 and *p* = 0.0474, respectively).

### Reoperations and complications

Among with full access to rehabilitation, reoperation and complication data was available for 68 (98.6%) patients. In terms of reoperations, the most common reoperation was the removal of hardware, performed in 10 (14.5%) patients (Table 4). Two patients failed MLKR and later underwent total knee arthroplasty (TKA) at 572 days post‐operatively and 1522 days post‐operatively. Arthrofibrosis was the most common complication, seen in 12 (17.6%) patients, followed by symptomatic or failed hardware in 9 (13.2%) patients.

**Table 4:** Comparison of reoperations between patients with full and limited access to rehabilitation.

Reoperations	Full rehabilitation access (*n* = 68)	Limited rehabilitation access (*n* = 14)
Closed manipulation under anaesthesia	7 (10.1%)	1 (7.1%)
Concomitant or open manipulation under anaesthesia	6 (8.7%)	0
Hardware removal	10 (14.5%)	0
Irrigation and debridement	3 (4.3%)	1 (7.1%)
Total knee arthroplasty	2 (2.9%)	1 (7.1%)
Revision LCL reconstruction	4 (5.8%)	0

Abbreviation: LCL, lateral collateral ligament.

Among patients with limited access to rehabilitation, reoperation and complication data was available for all 14 (100%). Two (14.3%) underwent reoperation. One (7.1%) underwent irrigation and debridement for septic arthritis, and one (7.1%) failed later undergoing TKA at 1223 days post‐operative (Table 4).

## DISCUSSION

In this study, patients with full access to rehabilitation demonstrated statistically significant improvements in PROMs using the PROMIS pain and PROMIS physical function scales compared to those with limited access to rehabilitation. PROMs using other scales like PROMIS mobility, MLQOL and Lysholm scores were similar between patients with full access to rehabilitation and patients with limited access to rehabilitation. Failure rates were relatively low in both patient groups.

While two of the PROMs evaluated here demonstrated statistically significant differences between those with full and limited access to rehabilitation, it is important to consider whether these differences are clinically meaningful. The minimal clinically important differences (MCIDs) for these PROMs have been established by prior studies, for PROMIS Pain the MCID is 8.9 and for PROMIS physical function the MCID is 11.3 [41]. Therefore, the statistical difference is likely not reflected as a noticeable difference in patient outcomes. Considering no differences were found among the other PROMs evaluated, the findings of this study indicate that there is no clinically meaningful difference in outcomes between MLKI patients with limited versus full rehabilitation access.

Management of MLKIs is complex and requires consideration of multiple stages, including preoperative, operative and post‐operative courses. Makaram et al. noted that a goal of future research should be to create expert consensus on rehabilitation protocols for MLKI reconstruction patients, noting that this is currently lacking [27]. Especially because MLKI patients have demonstrated deterioration of knee function over time following surgery [21]. Optimizing every stage of MLKR, including post‐operative course, will be important to try to maximize and maintain outcomes. One of the challenges of post‐operative rehabilitation for these patients is balancing the goals of stability and mobility. Early mobility is proposed to help prevent arthrofibrosis [32], whereas immediate immobility is thought to promote stability [11]. Other questions of post‐operative rehabilitation include the progression of weight‐bearing and the type of bracing. To our knowledge, this is the first report of how access to rehabilitation affects patient‐reported outcomes in MLKI reconstruction. Patients with full access to rehabilitation started their protocols immediately post‐operative, whereas the limited access group often waited multiple weeks to begin their protocols; our findings suggest that early initiation of rehabilitation and full access to resources are not associated with meaningful improvement of PROMs in MLKI patients.

This comparison of limited versus full access to rehabilitation subject groups touched on several aspects of post‐operative rehabilitation that currently lack consensus, one of which is early versus delayed mobility. In this study, the full‐access rehabilitation group began working on ROM earlier than the limited access group, which had to wait until the 2‐week post‐operative clinic visit to use a hinged brace. Angelini et al. and Mook et al. report superior results post‐operatively with a short period of immobilization (<2 weeks) followed by an early ROM and recommend against prolonged immobilization [1, 30]. Specifically, Mook et al. demonstrated that patients who underwent prolonged immobility suffered more severe flexion and extension deficits as well as increased joint instability in all directions compared with those patients undergoing earlier mobility [30]. Knee immobilizer use may have contributed to the statistically worse physical function scores seen in the limited access group of patients, but it is likely not enough to be noticeable for the patients. Bracing is an important consideration because the type of bracing affects the progression of mobility. Hinged knee braces can be used to facilitate safe early motion [40], whereas the knee immobilizers used by the limited access group for the first 2 weeks post‐operatively limit motion. The patients with full access to rehab were able to work on ROM earlier as a result of their immediate access to a hinged knee brace. Multiple studies recommend the use of a hinged knee brace to facilitate controlled, early mobility post‐operatively [20, 24]. Conversely, this study demonstrated that PROM mobility scores were similar between the two groups in this study despite the difference in bracing. The rehabilitation recommendation with the most consensus is the stratification of weight‐bearing status (as well as the timing of weight‐bearing progression) based on the severity of the injury, including which ligaments are involved [6, 10, 23, 25, 26]. In this study, patients with earlier mobility, immediate use of a hinged knee brace, and more control stratification of weight‐bearing did not noticeably outperform those who temporarily used a knee immobilizer, with more variable weight‐bearing stratification.

Patients with full access to a regimented rehabilitation protocol also consistently utilized cryotherapy post‐operatively. The use of external cooling to a surgical site can help with pain control and is recommended following many orthopaedic procedures [17, 46]. Use of a cooling and compression device following knee arthroscopy decreased prostaglandin E_2_ levels, which indicates reduced inflammation [39]. The PROMs evaluated in this study were taken at least 1 year post‐operatively, so there is no insight into how cryotherapy in the immediate post‐operative period may have influenced their pain during that time. It is possible that consistent cryotherapy use may have improved immediate postop pain and had an anti‐inflammatory effect. However, the effects of consistent cryotherapy use did not contribute to any clinically meaningful changes in outcome at longer‐term follow‐up.

Rehabilitation can influence more than just functional recovery following reconstructive surgery; it affects patient pain as well. The statistically different PROMIS Pain scores identified here were not large enough to reflect clinical differences. On the other hand, Miller et al. found that in patients undergoing ACL reconstruction, those who had at least 15 physical therapy sessions had less pain than those attending less than 9 sessions [29]. Importantly, pain is a subjective measure despite the use of standardized surveys like PROMIS Pain. Pain can also be affected by several external factors, like the physical demands of a patients' day‐to‐day life. It is therefore possible that differences in pain are impacted by more than the difference in rehabilitation access between MLKI patients analyzed here. This study demonstrates that patients with limited access to rehabilitation resources likely did not experience noticeable differences in pain post‐operatively.

Despite the lack of clinically meaningful differences based on rehabilitation access identified here, an exploration of how to improve patient post‐operative experiences and outcomes regardless of access to care is valuable. Supervision of and compliance with rehabilitation go hand‐in‐hand. For patients with limited access to in‐person, supervised physical therapy and at‐home rehabilitation may provide a more accessible option. However, the efficacy of unsupervised rehabilitation done at home relies on the consistency and determination of the individual patient [13]. Patients with lower health literacy or less time to dedicate to home rehabilitation may have a more difficult time committing to their rehabilitation protocols. For patients vulnerable to poor access to post‐operative physical therapy, providing patient education reinforcing the importance of rehabilitation may be a good option. This may be the role of the surgeon in patients identified to be susceptible to poor access. Ideally, patients could use this tool to increase their consistency with physical therapy despite their limited ability to attend supervised or in‐person rehabilitation sessions. Grant et al. demonstrated that ACL‐R patients who underwent home physical therapy had a higher proportion of patients with acceptable flexion and extension ROM compared with the supervised therapy group [15]. Unsupervised home therapy has also proved to have good outcomes for patients undergoing total joint arthroplasties [5, 38]. Thus, future studies may further contribute to this topic in the MLKI literature by investigating how providing patient education on the importance of post‐operative rehabilitation as well as resources on implementing home therapy to augment their supervised therapy affects patient outcomes.

In establishing post‐operative courses for patients, the surgeon may have to cater to differences in access to care to optimize outcomes for each patient. While access to rehabilitation during the inpatient post‐operative course can be dependent on staffing and availability, these outcomes may see improvement if further collaboration between the operative team and rehabilitation team in these settings is encouraged. Patient education provided by the operative team on the importance of rehabilitation and ways to engage in physical therapy at home may also help to improve outcomes for these patients. Emphasis on partnership between occupational and physical therapists, surgeons, and patients may be the first step that the operative team can take.

This study has important limitations. Retrospective collection of data makes these findings subject to potential biases. Furthermore, the small size of the comparison group reduces the statistical power and generalizability of these findings. As is the case with all MLKI studies, there is significant variability in the injury severity and pattern. These groups were also not similar at baseline in terms of injury severity, creating an important confounding factor. There was also a considerable disparity in loss‐to‐follow‐up between groups, which further limits this analysis. Furthermore, pre‐operative PROMs were unable to be assessed due to the traumatic nature of the injury, often requiring acute surgical treatment. The use of assessments over the phone also introduces potential biases that cannot be controlled. Several potential confounding factors could not be controlled, including patient comorbidities and associated injuries. This study also did not collect data on the number or type of rehabilitation or physical therapy sessions attended by each patient, presenting another major limitation. We, therefore, could not stratify our results by the amount of physical therapy sessions, time spent in supervised rehabilitation or take therapist expertise into account. The quality and quantity of the rehabilitation and therapy of the limited access group were not assessed. Future studies on this topic should include this kind of data, which would better allow us to identify if there is a certain amount or kind of rehabilitation necessary to meet ideal outcomes.

## CONCLUSION

Patients who underwent MLKI reconstruction with limited access to rehabilitation demonstrated statistically worse PROM PROMIS pain and physical function scores than those with full access to rehabilitation in the short term. Importantly, these differences likely do not reflect clinically meaningful changes. Other PROMs like Lysholm, PROMIS mobility and MLQOL scores were similar between patients with and without full access to rehabilitation. PROMs in MLKI patients are similar regardless of access to rehabilitation.

## AUTHOR CONTRIBUTIONS

**George F. Rick Hatch 3rd**: Conceptualization; methodology; supervision. **Joseph N. Liu**: Conceptualization; methodology; supervision. **Shane S. Korber**: Conceptualization; methodology; formal analysis; investigation; writing—original draft; writing—review and editing. **Ioanna K. Bolia**: Conceptualization; methodology; formal analysis; investigation; writing—original draft; writing—review and editing. **Neilen Benvegnu**: Conceptualization; formal analysis; investigation. **Amir Fathi**: Formal analysis; investigation; writing—original draft. **Samantha A. Solaru**: Formal analysis; investigation. **Cailan L. Feingold**: Writing—original draft; writing—review and editing. **Eric H. Lin**: Writing—original draft; writing—review and editing.

## CONFLICT OF INTEREST STATEMENT

Joseph N. Liu reports a relationship with Stryker Orthopaedics that includes: speaking and lecture fees. Joseph N. Liu reports a relationship with Innocoll Biotherapeutics NA Inc that includes: travel reimbursement. George F. Rick Hatch 3rd reports a relationship with Arthrex, Inc that includes: paid consultant and paid presenter or speaker. The remaining authors declare no conflicts of interest.

## ETHICS STATEMENT

All procedures performed in studies involving human participants were in accordance with the ethical standards of the institutional and/or national research committee and with the 1964 Helsinki Declaration and its later amendments or comparable ethical standards. The study was approved by the Institutional Review Board of the University of Southern California (#HS‐17‐00301). Informed consent was obtained from all individual participants included in the study.

## Supporting information


**Supplemental Table**: Comparison of PROM scores by KD class.

## Data Availability

The participants of this study did not give written consent for their data to be shared publicly, so due to the sensitive nature of the research, supporting data is not available.
