# Supplementary material for: Multiligament knee injury patients with limited access to post‐operative rehabilitation exhibit similar patient‐reported outcomes: A retrospective cohort study
Source: J Exp Orthop. 2025 Apr 22;12(2):e70245. doi: 10.1002/jeo2.70245 (PMC12012577; doi:10.1002/jeo2.70245)
Supplement: Supplementary file 1 — Supplemental Table: Comparison of PROM scores by KD class. [file JEO2-12-e70245-s001.docx]

| **PROM** | **KD Class** | **Full Access** | **Limited Access** | **p-value** |
| --- | --- | --- | --- | --- |
| PROMIS Pain | KD 1 | 52.4+9.9 | 60.3+1.2 | 0.1574 |
|  | KD 2 | 54.0+8.9 | None |  |
|  | KD 3 | 49.9+10.8 | 58.3+8.4 | **0.0239** |
|  | KD 4 | 54.4+9.2 | none |  |
| PROMIS Physical Function | KD 1 | 47.3+12.4 | 43.6+2.7 | 0.1964 |
|  | KD 2 | 45.0+7.0 | None |  |
|  | KD 3 | 46.6+9.8 | 38.1+9.6 | **0.0474** |
|  | KD 4 | 42.3+13.5 | None |  |
| PROMIS Mobility | KD 1 | 45.8+10.5 | 39.7+7.4 | 0.2909 |
|  | KD 2 | 42.9+8.2 | None |  |
|  | KD 3 | 46.5+9.6 | 39.6+9.9 | 0.0679 |
|  | KD 4 | 42.3+11.2 | None |  |
| MLQOL Physical Impairment | KD 1 | 40.0+27.6 | 57.0+5.3 | 0.2463 |
|  | KD 2 | 32.1+15.2 | None |  |
|  | KD 3 | 39.4+24.7 | 37.7+28.8 | 0.8571 |
|  | KD 4 | 53.4+13.5 | None |  |
| MLQOL Emotional Impairments | KD 1 | 46.9+33.7 | 63.9+10.0 | 0.5942 |
|  | KD 2 | 43.7+22.6 | None |  |
|  | KD 3 | 44.0+27.2 | 47.1+27.4 | 0.8571 |
|  | KD 4 | 60.7+14.7 | None |  |
| MLQOL Activity Limitations | KD 1 | 38.0+29.3 | 50.7+20.4 | 0.5942 |
|  | KD 2 | 29.0+16.3 | None |  |
|  | KD 3 | 38.5+25.6 | 43.9+25.6 | 0.5690 |
|  | KD 4 | 38.7+29.3 | None |  |
| MLQOL Social Involvement | KD 1 | 41.5+29.8 | 54.2+11.0 | 0.1846 |
|  | KD 2 | 37.9+22.4 | None |  |
|  | KD 3 | 44.1+28.9 | 50.0+21.8 | 0.5093 |
|  | KD 4 | 50.6+29.3 | None |  |

**Supplemental Table**: Comparison of PROM scores by KD class
